# Ectomycorrhizal Fungal Community and Ascoma Production in a Declining *Tuber borchii* Plantation

**DOI:** 10.3390/jof9060678

**Published:** 2023-06-15

**Authors:** Francesca Ori, Marco Leonardi, Federico Puliga, Enrico Lancellotti, Giovanni Pacioni, Mirco Iotti, Alessandra Zambonelli

**Affiliations:** 1 Department of Life, Health and Environmental Sciences, University of L’Aquila, Via Vetoio, Coppito 1, 67100 L’Aquila, Italy; francesca.ori.agronomist@gmail.com (F.O.); marco.leonardi@univaq.it (M.L.); giovanni.pacioni@univaq.it (G.P.); 2 Department of Agricultural and Food Sciences, University of Bologna, Viale Fanin 44, 40127 Bologna, Italy; federico.puliga2@unibo.it (F.P.); alessandr.zambonelli@unibo.it (A.Z.); 3 Sardinian Truffle Association, Loc. Santa Sofia, 09090 Laconi, Italy; enrlanc@gmail.com

**Keywords:** *Pinus pinea*, oak, *Tuber maculatum*, *Tuber rufum*, rDNA ITS region, ascoma production, competition

## Abstract

*Tuber borchii* is an edible ectomycorrhizal mushroom of considerable economic value. Its cultivation has become popular in recent years, but there are few studies on the factors affecting its productivity. In this work, the ascoma production and the ectomycorrhizal (ECM) community of a *T. borchii* plantation, established in an intensive farming area where this truffle is not naturally present, were studied. *Tuber borchii* production drastically declined from 2016 to 2021, and ascomata of other *Tuber* species (*T. maculatum* and *T. rufum*) were found from 2017. Molecular characterization of ectomycorrhizae carried out in 2016 identified 21 ECM fungal species, of which *T. maculatum* (22%) and *Tomentella coerulea* (19%) were the most abundant. *Tuber borchii* ectomycorrizae (16%) were almost entirely confined to the fruiting points. The diversity and structure of the ECM community on *Pinus pinea* were significantly different from those observed on hardwood trees. The obtained results suggest that *T. maculatum* (a native of the study site) tends to replace *T. borchii* through a mechanism of competitive exclusion. Although *T. borchii* cultivation is possible in suboptimal environments, particular care should be taken to limit competition with ECM fungi more suitable for local conditions.

## 1. Introduction

Edible ectomycorrhizal mushrooms (EEMMs) are represented by about 1000 species within the ectomycorrhizal (ECM) fungi group [1]. EEMMs are spread across many Ascomycota and Basidiomycota genera and can form different epigeous or hypogeous reproductive structures [2]. Most of them are obligate symbionts and need a host plant to complete their life cycle [3].

The cultivation of EEMMs is based on the production of mycorrhized seedlings in a nursery and their plantation in suitable soils with optimal climatic conditions. So far, spores have been the most commonly used inoculum source for root colonization, but mycelium can also be successfully applied for some EEMMs. Some species in the *Tuber*, *Rhizopogon*, *Suillus*, and *Lactarius* genera have been reasonably well cultivated using seedlings inoculated with spores and/or pure cultures [4,5,6]. Cultivation of other EEMMs, such as *Boletus edulis* sensu lato (“Porcini”), encountered some problems because of the quick replacement of its ectomycorrhizae by other ECM fungi once the seedlings were transplanted in the field [7]. Among Ascomycetes, valuable species of true (*Tuber* spp.) and desert (*Terfezia* spp.) truffles are successfully cultivated around the world [8,9]. Only *Tuber magnatum* Picco cultivation has been unsuccessful so far, although the first evidence of ascoma production in a truffle plantation established outside its natural range of distribution has been recently reported [10,11].

It is important to understand all the complex interdependencies between ECM fungi, host plants, and other microorganisms present in the soil to improve the cultivation of EEMMs. Moreover, ECM communities occurring in natural habitats are composed of many species, and their evolution is determined by the natural dynamics of competition [2]. When an EEMM species is introduced into the field through the planting of mycorrhized seedlings, it would be useful to know the composition of the native ECM community since a number of ECM species, better suited to the local conditions, could replace it [4,12].

Among valuable truffles, *Tuber borchii* Vittad. is one of the species with the highest ecological adaptability, and its cultivation is becoming popular in several countries [13]. Nevertheless, only two studies on ECM communities of *T. borchii* orchards (natural and cultivated) are available, and both were conducted in the same habitat suited to this truffle species [14,15]. *Tuber borchii* has always been considered an early-stage fungus that fruits early on young host plants [14]. Recently, Iotti et al. [16] reported the first production of *T. borchii* ascomata from plants inoculated with five pure cultures. This truffle plantation is located in an intensive farming area where *T. borchii* is not naturally present [17,18] and ECM host plants are mainly confined to private gardens or urban parks. In this study, we followed the production of *T. borchii* ascomata on the same plantation from 2016 to 2021 and characterized the ECM community in the year of peak production. The obtained results allowed us to confirm the preference of this truffle for young plantations and to increase the knowledge on the following: (i) the correlation between ascoma production and ectomycorrhiza distribution of *T. borchii*; (ii) the competition between *T. borchii* and the native ECM fungi; and (iii) the impact of the host species on *T. borchii* competitiveness.

## 2. Materials and Methods

### 2.1. Plantation Site

The *T. borchii* plantation was established on the University of Bologna farm in Cadriano (Granarolo dell’Emilia municipality, Emilia Romagna, Italy). It is located in a typical rural landscape of the Po Valley, dominated by arable lands and orchards. The area is suited to some *Tuber* species, such as *T. magnatum*, *T. maculatum* Vittad., *T. brumale* Vittad., and *T. rufum* Picco, but *T. borchii* has never been found [17,18]. The ground is flat with an elevation of 26 m a.s.l. and characterized by a loamy soil (36% sand, 41% silt, 23% clay) with a pH in H_2_O of 7.5, organic matter 1.6% and both total and active limestone are less than 1%. The climate is temperate and sub-continental, with marked seasonal variations in both temperature and precipitation; minimum and maximum temperatures are in January and July, respectively, while rainfalls are highest in April and November and lowest in July (Figure S1) [19].

The seedlings of four ECM host species (*Pinus pinea* L., *Quercus pubescens* Willd., *Q. robur* L., and *Corylus avellana* L.) were inoculated with mycelium from five *T. borchii* strains and planted in autumn 2007 and 2008 in an area covering about 530 m^2^. Plants of the same species colonized with the same strain were grouped together and separated by guard rows of non-inoculated *C. avellana* seedlings, according to the plantation design reported in Figure S2. Detailed information on the *T. borchii* strains used for mycorrhization, inoculum preparation, features, and management of the plantation was reported by Iotti et al. [16].

### 2.2. Ascoma Collection

*Tuber borchii* ascomata were collected using our trained dogs between January and April, from 2016 (data reported by Iotti et al. [16]) to 2021. Collection time, weight, and position were recorded for each ascoma, except the year 2019, when fruiting bodies were harvested by a professional truffle hunter and only the ascoma weight was recorded. All fruiting bodies were identified using morphological and molecular methods [20,21] and deposited in the herbarium of the “Centro di Micologia” (Bologna University). Molecular identification of *Tuber* ascomata was carried out by using species-specific primers [22,23].

### 2.3. Ectomycorrhizal Sampling

Root sampling was carried out during the *T. borchii* fruiting period (February and March 2016) in six surveys at 10- to 15-day intervals. In total, 29 samples (6 cm in diameter and 15 cm deep) were collected in the proximity of inoculated plants (Figure S2): 17 in the points where an ascoma was found (fruiting point samples, FP) and 12 in at least 40–50 cm far from the fruiting point (fruiting area samples, FA). An additional 12 samples were collected in proximity to non-inoculated hazels (non-inoculated tree samples, NT) (Figure S2). The 41 root samples were taken over the whole surface of the truffle ground in order to get a sample distribution as evenly as possible.

### 2.4. Molecular Identification of Ectomycorrhizae

The roots were isolated from the soil by careful washing with tap water and then examined under a stereomicroscope (×20). Each ectomycorrhiza was assigned a morphotype on the basis of its anatomo-morphological characters after Agerer [24] and to hardwood (mainly *Q. pubescens*) or pine based on their ramification type. The ECM colonization was evaluated by counting the number of ectomycorrhizae of each morphotype and host type in all the root samples and expressing the results as a percentage of the total number of ectomycorrhizae examined for the whole community, host plant, or sample type.

One to three ectomycorrhizae from each morphotype and sample were selected for molecular characterization. These were vortexed for 1 min in 1.5 mL tubes containing 500 μL of sterile water, spun for 2 min at 13,000× *g* to remove any soil particles, transferred into a new 1.5 mL tube, and stored at −80 °C. Molecular characterization was performed by applying the direct PCR technique as described by Iotti and Zambonelli [25]. Fragments of the ectomycorrhizal mantle were amplified by a SimpliAmp Thermal Cycler (Applied Biosystem) in 50 μL volume reactions using the primer pair ITS1F and ITS4 [26,27] at a concentration of 400 nM each. Then, 40 μg of bovine serum albumin was added to compensate for PCR inhibitors. The cycling parameters were: 8 min of initial denaturation at 95 °C; 35 cycles of 94 °C for 30 s, 55 °C for 30 s, and 72 °C for 1 min; and final extension step of 72 °C for 10 min. PCR products were visualized through 1.5% agarose gel electrophoresis stained with ethidium bromide.

PCR products were purified using the QIAquick PCR Purification Kit (Qiagen, Milan, Italy) and then sequenced on both strands with the primers ITS5 and ITS4 [26] at the Eurofins Genomics sequencing service (Vimodrone, Milan, Italy). Sequences were edited and aligned using BioEdit [28] and then regarded as belonging to operational taxonomic units (OTUs) following the 97% threshold values of sequence similarity. OTUs were identified based on the comparison with the sequences deposited in the GenBank database (http://blast.ncbi.nlm.nih.gov/Blast.cgi, accessed on 2 May 2023) using the BLASTN search [29] and by applying the following criteria: sequence similarity ≥ 97%, identification to species level; sequence similarity 95–97%, identification to genus level; sequence similarity of ≤95%, identification to family or order level. Sequences obtained in this study were deposited in the GenBank database with the following accession numbers: MH681172 to MH681192, MG973282 to MG973285 and OQ861263 (Table S1). GenBank accession numbers for *T. borchii* ectomycorrhizae can be retrieved from Bonuso et al. [30], who reported the genetic characteristics of the inoculated mycelial strains.

Since ectomycorrhizae of *T. borchii*, *T. maculatum*, and *T. rufum* [23,31] are morphologically difficult to distinguish and all these truffles were found in the study site, additional molecular analyses were needed to avoid bias in assessing the structure of ECM community. When *T. borchii*-like ectomycorrhizae were present in a root sample, species-specific PCRs were carried out on 10–20 additional tips for each sample. Mantle fragments of the same ectomycorrhiza were used as a target for multiplex and simple PCRs to discriminate *T. maculatum*/*T. borchii* [22] and *T. rufum* [23], respectively.

### 2.5. Statistical Analysis

The diversity of each ECM community (whole, hardwoods and pine, FP, FA, and NT samples) was assessed and represented using a dominance-diversity curve and species richness^®^, Pielou (J), and Shannon (D) indices. Root samples containing ectomycorrhizae of both hardwoods and pine (4 FP samples in total) were split into two subsamples containing ectomycorrhizae of a single host type to allow statistical computation. Significant differences between the means of diversity indices of hardwoods vs. pine and FP vs. FA communities were determined by a one-way ANOVA followed by Tukey’s test (*p* < 0.05). Multivariate analyses were also performed to evaluate the effect of host type (hardwoods vs. pine) and sampling position (FP vs. FA) on the ECM community. The similarity values between communities were calculated using the Bray–Curtis dissimilarity index. The species dissimilarity matrix generated by Bray-Curtis coefficients was analyzed using the Constrained Analysis of Principal Coordinates (CAP) [32] and Adonis test. NT samples were not considered for ANOVA and CAP analyses because being collected in the proximity of non-inoculated trees, they are not comparable with FP and FA samples. Statistical analyses were performed using R 2.9 software (R Development Core Team, 2009) with the ‘VEGAN’ package v. 11.1–4 [33].

## 3. Results

### 3.1. Ascoma Production

**Figure 1:**
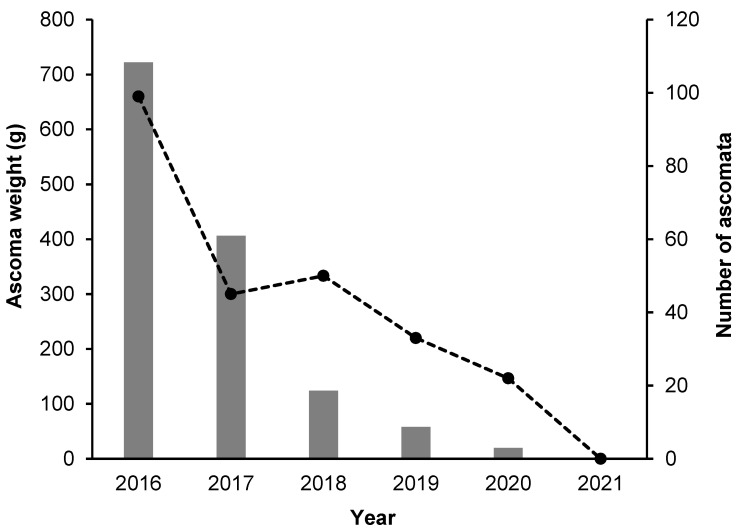
Number and weight of ascomata collected in the study site (2016–2021). Columns and black circles indicate the weight and the number of ascomata, respectively.

The production of *T. borchii* over 6 years was 249 ascomata with a total weight of 1330 g. It decreased steadily from 2016 (722 g) to 2020 (20 g), and in 2021, no *T. borchii* ascomata were found (Figure 1). The average weight of ascomata also decreased from 2016 to 2020, as follows: 7.3 g in 2016, 9.0 g in 2017, 2.5 g in 2018, 1.8 g in 2019, and 0.9 g in 2020. The position of each ascoma is reported in Figure S3, except for those of the year 2019. Ten ascomata of *T. maculatum* (4 in 2017, 3 in 2020, and 3 in 2021) with a total weight of less than 10 g and four of *T. rufum* (2020) with a weight of less than 1 g each were also found during the same period. Here, 8 out of 10 *T. maculatum* ascomata were found in proximity to plants where ectomycorrhizae of this truffle were also found in 2016 (plants 2, 12, 18, 19, 34, and 62), as well as one ascoma of *T. rufum* (plant 55) (Figures S2 and S3). Only two ascomata of *T. maculatum* were found near inoculated plants (plants 24 and 34), while all other ascomata of *T. maculatum* and *T. rufum* were found near non-inoculated hazels.

### 3.2. Whole ECM Fungal Community

**Figure 2:**
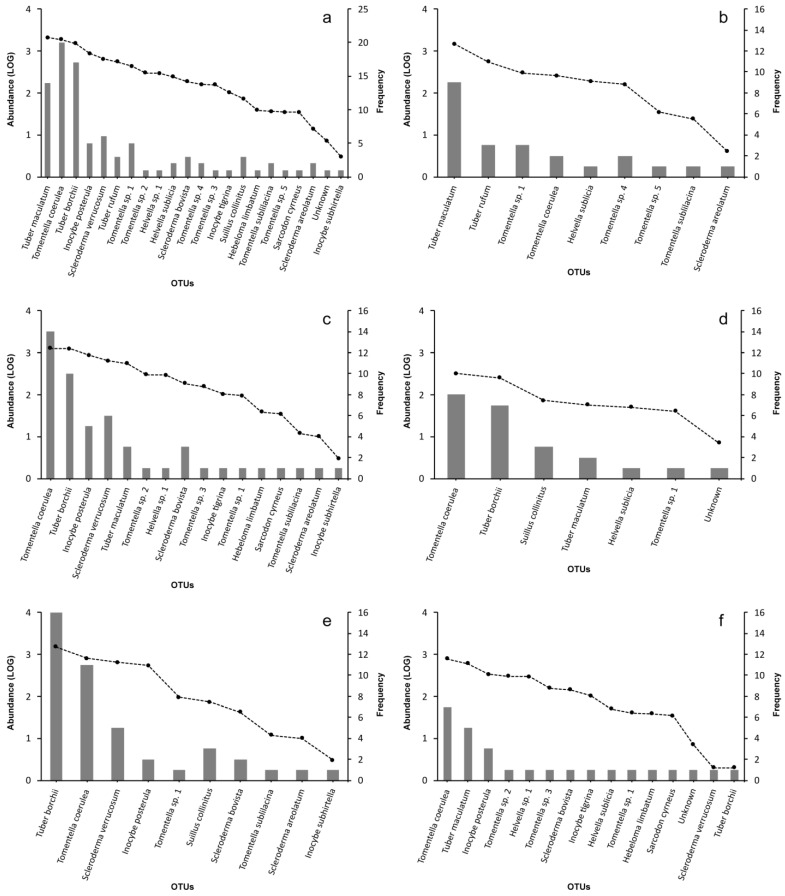
Dominance-diversity curves of ECM community in 2016: whole community (**a**); non-inoculated tree samples (**b**); hardwoods (**c**) and pine (**d**) root samples; fruiting points (**e**) and fruiting area (**f**) samples. The black circles and bars indicate the relative abundance (Log values) and the incidence (n. of root samples) for each OTU, respectively.

A total of 9592 ectomycorrhizae were counted in the 41 root samples collected for analyses. Based on molecular and morphological analyses, ectomycorrhizae were assigned to 21 OTUs (5 ascomycetes and 16 basidiomycetes) and one molecularly unidentified morphotype (Table 1, Figure 2a). All the *T. borchii* ITS sequences obtained in this study were identical to those of the strains used to inoculate the trees (clade 1 after [30]). About 400 ectomycorrhizae with a *T. borchii*-like morphology from 33 root samples were subjected to species-specific PCRs to discriminate among the three truffle species present in the study site. Ectomycorrhizae of *T. borchii*, *T. maculatum,* and *T. rufum* were never found to co-occur in the same root sample (Table S2).

The dominance-diversity curve (Figure 2a) showed that three OTUs were dominant, while the other 19 OTUs accounted in total for less than 44% of ectomycorrhizae (Table 1 and Table S1). The genus with the highest OTU richness was *Tomentella* (7), followed by *Scleroderma* (3), *Inocybe* (3), and *Tuber* (3). *Tuber maculatum* was the most abundant OTU with 22% of ectomycorrhizae, whereas the most frequent OTU was *Tomentella coerulea,* found in 20 out of 41 root samples. The inoculated species *T. borchii* accounted for 16% of ectomycorrhizae in 39% of samples. The values of the Shannon and Pielou indexes for the whole ECM community were 2.18 and 0.74, respectively (Table 1). Intraspecific variability of ITS1-5.8S-ITS2 sequences was found in *Tomentella coerulea*, *Tuber maculatum,* and *T. rufum* (1.90%, 1.07%, and 0.35% of nucleotide divergence, respectively), while single ITS haplotypes were sequenced for the other OTUs. No accessions with ITS1-5.8S-ITS4 sequence identity > 97% were found for 3 OTUs (*Tomentella* sp. 3, *Tomentella* sp. 4, *Tomentella* sp. 5) after a Blastn search against the GenBank database (access date: 2 May 2023).

### 3.3. Host Type (Pine vs. Hardwoods)

**Figure 3:**
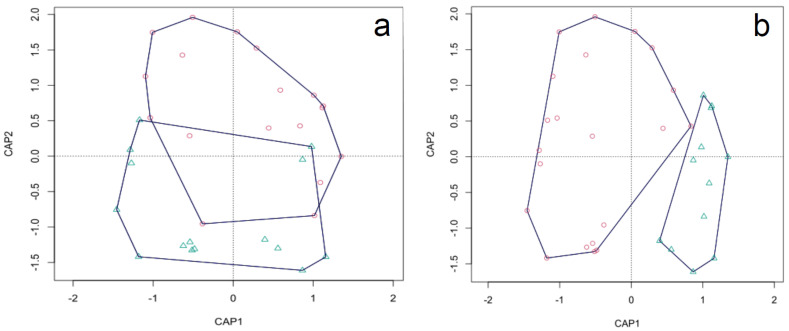
Constrained Analysis of Principal Coordinates: pine (blue triangles) and hardwood (red circles) root samples (**a**); fruiting point (red circles) and fruiting area (blue triangles) samples (**b**).

The number of ectomycorrhizae assigned to pine (798) and hardwoods (5808) in FP/FA root samples were very different. Here, 16 and 7 OTUs were found on hardwood and pine roots, respectively (Table 1, Figure 2c,d), 4 of which were common to both host types. *Tomentella coerulea* was the most abundant species on both hardwoods and pine, while *Tuber borchii* accounted for 32% and 21% of pine and hardwood ectomycorrhizae, respectively. Considering the other truffle species, *T. maculatum* occurs with 9.5% ectomycorrhizae on hardwood and 7.1% on pine roots. All diversity indices of the ECM community on pine were significantly lower than those on hardwoods (R, D, J = *p* < 0.01). Additionally, the ECM community composition was significantly divergent between the host types after the Adonis test (*p* < 0.01). The differences in α diversity between host communities were confirmed by the different positions of pine and hardwood samples in the ordination plot (Figure 3a). The host type explained 6.1% of the total variability (*p* = 0.015) of the ECM community.

### 3.4. Sampling Position (FP vs. FA)

FP samples contained a higher number of ectomycorrhizae (3718 vs. 2888) but fewer OTUs (10 vs. 15) with respect to FA samples (Table 1 and Figure 2e,f). No significant differences were found in any of the diversity indices between the FP and FA samples (Table 1). On the contrary, ECM community composition differed significantly among samples collected under *T. borchii* fruiting bodies or in the surrounding area (*p* < 0.01) (Figure 3b). The distribution of samples in the ordination plot clearly shows the separation between FP and FA fungal communities, which could be due to the uneven distribution of *T. borchii* and *T. maculatum* ectomycorrhizae (Table S2). Indeed, *T. borchii* was the most abundant (40%) and frequent (16 out of 17; 94%) species in FP samples, but it was found in only one FA sample (1 out of 12; <0.1%). On the contrary, *T. maculatum* was never detected in any FP sample, but it was the dominant species (abundance 21%, frequency 42%) in FA samples together with *T. coerulea* (abundance 27%, frequency 58%). The sampling position explained 11.9% of the total variability (*p* = 0.001) of the ECM community.

### 3.5. ECM Fungal Community in Proximity to Non-Inoculated C. avellana Seedlings (Samples NA)

Ectomycorrhizae collected under non-inoculated *C. avellana* seedlings were 2986 sorted in 9 OTUs. ECM community of NT samples was mostly represented by *T. maculatum* (49%) and *T. rufum* (19%), followed by *Tomentella* sp. 1 (10%), *T. coerulea* (9%), and *Helvella sublicia* (6%) (Figure 2b). *Tuber borchii* was never detected in NA root samples. Diversity indices are reported in Table 1.

## 4. Discussion

Characterization of the ECM community in truffle production areas has been carried out for all the most valuable *Tuber* species, such as *T. magnatum* [34,35], *T. borchii* [14,15], *T. aestivum* [36,37], *T. macrosporum* [38], and *T. melanosporum* [39,40,41,42]. Usually, these studies were conducted in marginal areas where the target truffle grows naturally, and, in the case of artificially established plantations, mycorrhizal plants were obtained by spore inoculum. In this study, the ECM community was analyzed in a truffle plantation established using mycelial infected seedlings planted in an intensive farming area where the target truffle is not present.

Among the ECM genera commonly found in the truffle areas [43], *Tomentella*, *Inocybe*, *Scleroderma,* and *Tuber* accounted for more than 70% of the OTUs of the studied ECM community, while *Sebacina* spp. were absent, and the diversity of Pezizalean ECM fungi was unexpectedly low. Indeed, Sebacinales and Pezizales (particularly Pyronemataceae spp. and Pezizaceae spp.) have been commonly found both in *T. borchii* and *T. magnatum* truffle grounds [15,25,34].

Excluding truffle species, at least 10 other OTUs were already found by studies carried out in typical truffle habitats. In particular, *Tomentella coerulea*, the most common OTU in the study site, as well as *Inocybe tigrina*, *I. subhirtella*, and *Suillus collinitus* were also found in littoral sandy soils, about 100 km from the study area, where *Tuber borchii* is naturally present [15]. *Tomentella* sp. 1, *Tomentella* sp. 2, and *T. sublilacina* were also present in French *T. melanosporum* and/or Italian *T. magnatum* grounds [35,41], *Scleroderma verrucosum* in Italian and French *T. melanosporum* grounds [39,41], and *S. areolatum* in an Italian *T. macrosporum* ground [38]. Lastly, *Helvella sublicia* was reported in a *T. aestivum* field in Poland [44]. The α diversity of the overall ECM community is in line with the values calculated for other cultivated truffle ground [37,38,39] and generally lower than those obtained in natural truffle ground [15,35,39,41].

Nine years after plantation, the abundant production of *T. borchii* ascomata was supported by about 25% of root colonization in the area occupied by the inoculated plants. This value was just below that estimated at planting time (30–40%). Preliminary analysis conducted by Iotti et al. [16] on the same plantation reported that *T. borchii* ectomycorrhizae reached 70% in some root samples and that even some non-inoculated hazels showed its ectomycorrhizae. In that study, the characterization of ectomycorrhizae was done exclusively based on morphological traits, while our analyses were conducted with the support of molecular techniques that allowed us to distinguish also among ectomycorrhizae with similar anatomy and morphology. In particular, *T. maculatum* and *T. rufum* ectomycorrhizae were morphologically indistinguishable from *T. borchii* ectomycorrhizae [14,23]. This shared morphology has misled Iotti et al. [16], who overestimated the ectomycorrhizae of *T. borchii* within the truffle plantation. These results stress the importance to apply in-depth molecular characterization of the ECM community in truffle plantation since many *Tuber* species have ectomycorrhizae with very similar mantles (round to epidermoid cells) and cystidia (awl-like type) [31]. Moreover, the cystidia can be absent depending on the ontogenetical stage of the ectomycorrhiza [45,46].

*Tuber borchii* ectomycorrhizae were almost entirely confined to the fruiting points, to the extent that they significantly affected the structure of the ECM community but not the α diversity indices. Similar behavior of this truffle was also observed in mixed pine-oak woods, where it naturally grows [15]. The absence of its mycorrhizae under the non-inoculated hazels in 2016 might be considered evidence of a low diffusion ability of the *T. borchii* extraradical mycelium under the environmental conditions of this study. This conflicts with the results obtained by Zambonelli et al. [14] who reported a high capacity of *T. borchii* to colonize non-inoculated plants of a *P. pinea* plantation established in a coastal area where this truffle naturally grows. Considering that the ectomycorrhizae of *T. borchii* were mainly located near their ascomata, the steady and rapid decline in truffle production after 2016 suggests that *T. borchii* survived at significant levels in the experimental plantation for just over 10 years. Since 2021, rare ectomycorrhizae of *T. borchii* could still be present somewhere in the truffle plantation, but, likely, they are not sufficiently abundant to support ascoma production. This production time frame as well as the movement pattern of the fruiting points within the truffle plantation over the years seem to be similar to those reported by Zambonelli et al. [14]. Overall, fruiting points are evenly distributed on the whole surface when the *T. borchii* trees are young and then decrease and move to the border, or openings of the plantation. In the study case, most of the ascomata collected from 2017 to 2020 were found in gaps left by dead plants, in the middle of the country lane separating the truffle plant rows, or along the external edges of the plantation. Zambonelli et al. [14] attributed the decline in *T. borchii* production to canopy closure and litter accumulation, as was the case in this study. Additionally, the constant reduction in ascoma size as a function of plantation age is noteworthy.

Two main contrasting factors may have contributed to accelerating or slowing down *T. borchii* decay at the study site. The first is represented by *T. maculatum,* which seems to be the main factor responsible for *T. borchii* ECM replacement through a mechanism of competitive exclusion. No specific studies on competition between these two truffle species have been carried out so far. Our results showed that *T. maculatum* was the only species among the most abundant OTUs (*Tomentella coerulea*, *Inocybe posterula*, *Scleroderma verrucosum*) whose ectomycorrhizae were never found in the same soil core as those of *T. borchii*. Most likely, *T. maculatum* first colonized the roots of non-inoculated hazels and then spread to the adjacent fruiting areas, replacing *T. borchii*. This was supported by the finding of *T. maculatum* ectomycorrhizae (e.g., plants 34, 45, and 15) and ascomata (plants 24 and 34) within the fruiting *T. borchii* areas. The difference in competitive ability between these truffle species could be due to the specific characteristics of the study site. *Tuber maculatum* is common in alluvial soils, whereas *T. borchii* is more frequent in sandy soils of littoral or Apennine woods [47]. The natural diffusion of *T. maculatum* was also shown in a recent study where the mycelia of *T. maculatum* were found to coexist with those of *T. magnatum* in more than 70% of the analyzed soil samples [43]. As with *T. magnatum*, *T. maculatum* seems to prefer older woods with a close canopy. Moreover, differences in soil features could also affect competition between *T. borchii* and *T. maculatum,* as has already been reported for other truffle species. For example, Ori et al. [48] suggested that the low content of organic matter and high soil pH seem to protect *T. melanosporum* from the aggression of *T. brumale*.

The second factor that may have affected the maintenance of *T. borchii* in the plantation is the host species. *Pinus pinea*, the most suitable host for *T. borchii*, is not common in the study area, and, likely, it finds less compatible ECM fungi than hardwoods. This would explain the significantly lower α diversity and the highly divergent composition of the ECM pine community, which could have favored the maintenance of *T. borchii*, slowing down its replacement by *T. maculatum* and the other native ECM fungi.

## 5. Conclusions 

This work provides new insights for improving *T. borchii* cultivation strategies. We demonstrated that it can also be successfully cultivated in sub-optimal environments, although particular care should be taken to maintain its ectomycorrhizae for as long as possible on truffle plants. In particular, adequate host species, planting distances, and agronomic practices must be adopted to promote its competitiveness with respect to some native ECM fungi that tend to replace it. In the study case, plant pruning, litter removal, or soil tillage might be investigated in order to restore *T. borchii* production and mycorrhization. Wider planting distances should also be adopted during orchard establishment to delay the canopy closure and, consequently, extend the fruiting period and increase the ascoma size. Moreover, the plantation of non-inoculated plants, like the hazels in our study, should be avoided because they may favor the entrance and diffusion of contaminants in the truffle plantation.

## Figures and Tables

**Table 1:** Diversity indices (species richness R, evenness J, and Shannon D) of the whole, host type, and sampling position ECM communities. Different letters in the same column and sample type indicate significant differences for *p* < 0.01.

Community Type	Root Samples (n)	Ectomyc. (n)	R	D	J
Whole	41	9592	22	2.176	0.739
Hardwoods	17	5808	16 a	2.155 a	0.777 a
Pine	15	798	7 b	1.483 b	0.762 b
Fruiting points (FP samples)	17	3718	10	1.543	0.770
Fruiting areas (FA samples)	12	2888	15	2.081	0.768
Non-inoculated trees (NT samples)	12	2986	9	1.538	0.517

## Data Availability

The raw data of this study are available from the corresponding author.

## Supplementary Materials

The following supporting information can be downloaded at: https://www.mdpi.com/article/10.3390/jof9060678/s1, Figure S1—Ombrothermic diagrams (1952 to 2014) for the station in the study site (Cadriano, Granarolo nell’Emilia, Bologna, Italy). The solid line indicates the mean monthly temperature; the dashed line indicates monthly rainfall; Figure S2—Scheme of the *T. borchii* plantation. Colored numbers indicate the type and position of root samples (red, FP—fruiting point samples; green, FA—fruiting area samples; blue, NT—non-inoculated tree samples). Inside the circle is reported the plant number in black. Different circle types indicate different host plants; Figure S3—Scheme of the plantation implemented with the fruiting position of *T. borchii* (asterisks: black 2016, red 2017, green 2018, violet 2020), *T. maculatum* (yellow triangles) and *T. rufum* (orange triangles). The fruiting position was not registered in 2019.
