# Supplementary material for: Ectomycorrhizal Fungal Community and Ascoma Production in a Declining Tuber borchii Plantation
Source: J Fungi (Basel). 2023 Jun 15;9(6):678. doi: 10.3390/jof9060678 (PMC10301055; doi:10.3390/jof9060678)
Supplement: Supplementary file 1 [file jof-09-00678-s001.zip › Fig. S2.pdf]

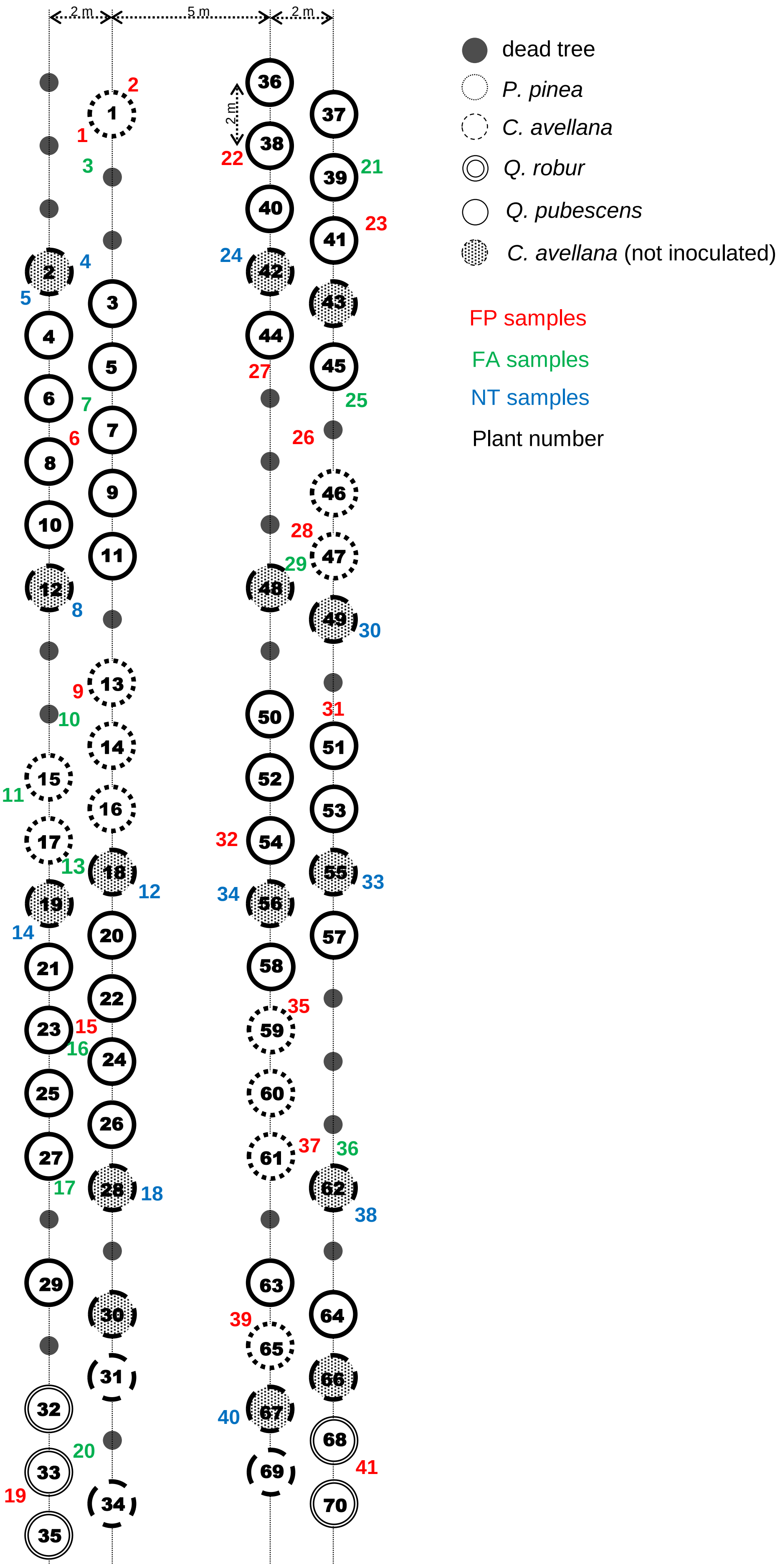

2 m
5 m
2 m
dead tree
P. pinea
C. avellana
Q. robur
Q. pubescens
C. avellana (not inoculated)
FP samples
FA samples
NT samples
Plant number
