# Supplementary figures and images for: Ectomycorrhizal Fungal Community and Ascoma Production in a Declining Tuber borchii Plantation
Source: J Fungi (Basel). 2023 Jun 15;9(6):678. doi: 10.3390/jof9060678 (PMC10301055; doi:10.3390/jof9060678)

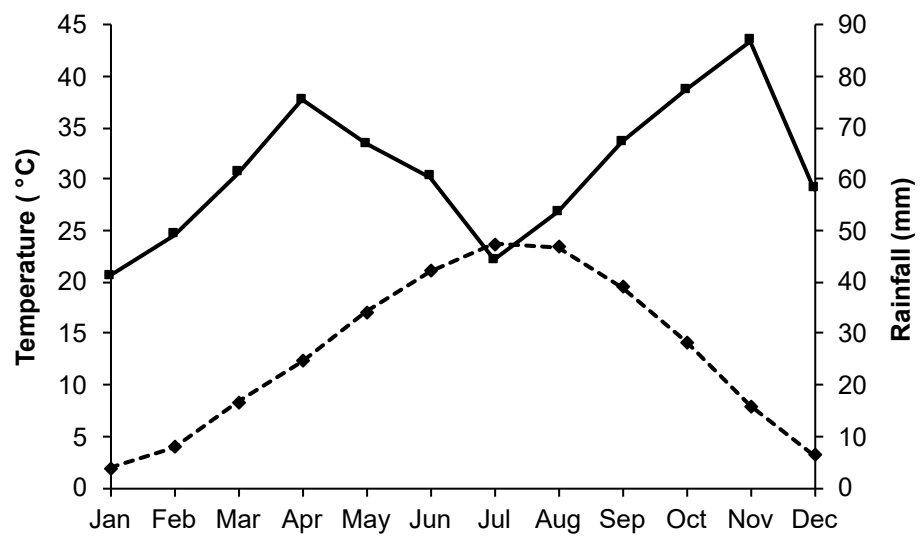

Supplement: Supplementary file 1 [file jof-09-00678-s001.zip › Fig. S1.pdf]

*T. borchii* fruiting points

2016 \*

2017 \*

2018 \*

2020 \*

▲ *T. maculatum* fruiting points

▲ *T. rufum* fruiting points

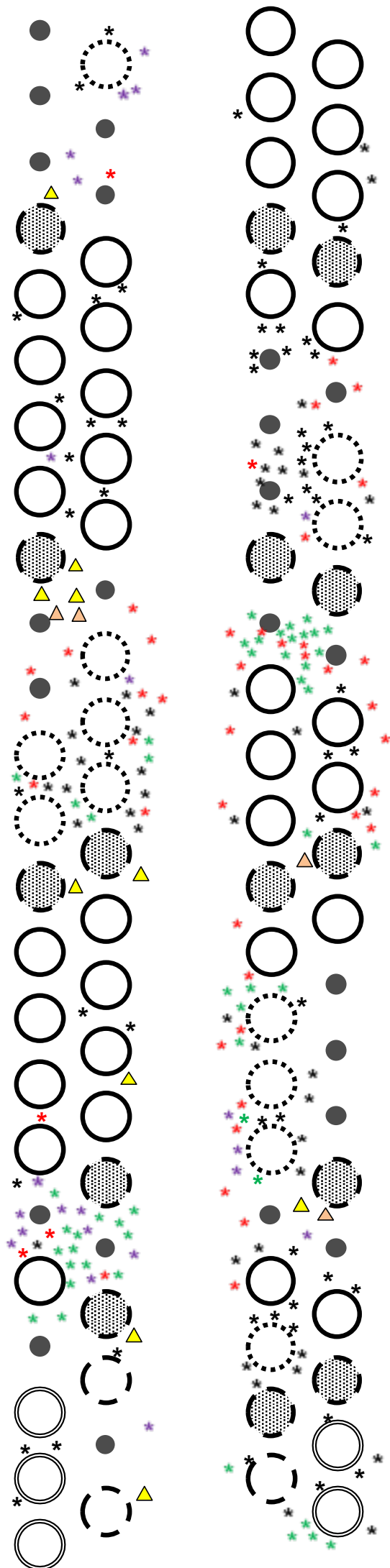

Supplement: Supplementary file 1 [file jof-09-00678-s001.zip › Fig. S3.pdf]
